# Comparison between preoperative and real-time intraoperative planning ^125^I permanent prostate brachytherapy: long-term clinical biochemical outcome

**DOI:** 10.1186/1748-717X-8-288

**Published:** 2013-12-17

**Authors:** Haim Matzkin, Juza Chen, Larissa German, Nicola J Mabjeesh

**Affiliations:** 1 Department of Urology, Tel Aviv Sourasky Medical Center, Sackler Faculty of Medicine, Tel Aviv University, Tel Aviv, Israel

**Keywords:** Prostate cancer, Brachytherapy, Implant technique, Biochemical failure

## Abstract

**Background:**

The purpose of the study is to evaluate the long-term clinical outcome through biochemical no evidence of disease (bNED) rates among men with low to intermediate risk prostate cancer treated with two different brachytherapy implant techniques: preoperative planning (PP) and real-time planning (IoP).

**Methods:**

From June 1998 to July 2011, 1176 men with median age of 67 years and median follow-up of 47 months underwent transperineal ultrasound-guided prostate ^125^I-brachytherapy using either PP (132) or IoP (1044) for clinical T1c-T2b prostate adenocarcinoma Gleason <8 and prostate-specific antigen (PSA) <20 ng/ml. Men with Gleason 7 received combination of brachytherapy, external beam radiation and 6-month androgen deprivation therapy (ADT). Biological effective dose (BED) was calculated using computerized tomography (CT)-based dosimetry 1-month postimplant. Failure was determined according to the Phoenix definition.

**Results:**

The 5- and 7-year actuarial bNED rate was 95% and 90% respectively. The 7-year actuarial bNED was 67% for the PP group and 95% for the IoP group (*P* < 0.001). Multivariate Cox regression analyses identified implant technique or BED, ADT and PSA as independent prognostic factors for biochemical failure.

**Conclusions:**

Following our previous published results addressing the limited and disappointing outcomes of PP method when compared to IoP based on CT dosimetry and PSA kinetics, we now confirm the long-term clinical, bNED rates clear cut superiority of IoP implant methodology.

## Background

Prostate brachytherapy has enjoyed an unparalleled resurgence in the last two decades. It represents the ultimate 3-D conformal therapy. Reported dose escalation far exceeds the best reported results of other radiation modalities. Current brachytherapy results have been obtained using a variety of planning and pre- and intra-operative calculations, of which no method was proven superior [1-3]. Historically, the prostate has been implanted using pre-planning (PP) dosimetric methods [2,4], where a planning transrectal ultrasound (TRUS) prostate volume study was done several weeks before the procedure, a treatment plan was conceived and on the day of the implant in the operating room the intraoperative patient positioning should mimic exactly the pre-implant study 3 dimensional cohorts. Several groups have described the potential and observed disadvantages of this method [5-7]. The evolution of TRUS and mainly the availability of sophisticated treatment planning computers, have evolved into the intraoperative planning (IoP) methodology and made better accuracy of intraoperative dosimetry and seed placement [1,8,9]. Intuitively, many brachytherapy centers have moved to the various IoP techniques, but with no clinical research data to substantiate this transition.

We had a unique opportunity since 1998 to compare in a prospective manner the two implant planning methods. Both treating physicians and the radiation physicists remained unchanged during the entire brachytherapy cohort analysis. Inclusion and exclusion criteria for treatment eligibility as well as treatment protocols remained unchanged during the entire period. Thus enabling us a fair comparison of the implant methods as the only variable changed along time. Previous reports of our group dealt with early post treatment results comparison: computerized tomography (CT)-based dosimetry calculations [10], urinary morbidity [5] and early prostate-specific antigen (PSA) kinetics following treatment [11]. Ultimately, clinical outcomes such as biochemical no evidence of disease (bNED) rate are of the utmost importance therefore we report our long-term PSA-based bNED rates for the groups.

## Methods

### Patients

The institutional review board of the Tel Aviv Sourasky Medical Center approved this study and waived informed consent requirements. A total of 1176 consecutive patients with biopsy-proven prostate cancer were treated with transperineal ^125^I-based permanent brachytherapy between June1998 and July 2011 in our center. Men with Gleason score ≤ 6, clinical stage T_1_–T_2_ and PSA < 20 ng/ml were given ^125^I brachytherapy as monotherapy targeting 100% isodose of 160 Gray (Gy). Men with Gleason score 7, of the same stage or PSA values were given a combination of ^125^I brachytherapy (100% isodose of 107 Gy) and external beam radiotherapy (EBRT), 45 Gy at a daily dose of 1.8 Gy. Androgen deprivation therapy (ADT) was given either to reduce gland size, if gland volume was above 55–60 cc, or in all men with Gleason 7 disease treated with combined radiation therapy. Duration of ADT ranged from 6 to 9 months. Of the entire cohort, 132 were treated in the initial period with the PP method, which was then switched to the IoP methodology by which the next 1044 consecutive patients were treated. Patient selection criteria and risk stratification for monotherapy vs. combined treatment were established at the start of our program, remained constant, and were regardless of the PP or IoP methodology. Patients were seen at 1, 3, 6, 9, 12 months and every 6 months thereafter. CT dosimetry, as suggested by the American Brachytherapy Society (ABS), was obtained at 1 month. Serum PSA was determined at each visit from month 3 onwards. Biochemical failure was defined using the Phoenix definition of a rise by 2 ng/mL or more above the nadir PSA [12].

### Brachytherapy planning methods

Methodology of PP and IoP techniques was described by several authors. In brief, in the PP, well described by Ragde and coworkers [4,13,14], a detailed ultrasound measurement and mapping was done in the lithotomy position some 4 to 6 weeks before operating day, and ultrasound data were sent to the radiation physicist to plan and choose the optimal dosimetry. At the day of treatment, the physicist prepared the needles with seeds and spacers in strict adherence to the preplan. In the operating room, all patients were placed in lithotomy position in an attempt to be identical to that placed at measurement, and care was taken to duplicate exact positioning in order to optimize dosimetry. Only then were needles inserted and seeds deployed according to the preplan. The IoP methodology described in detail by Stone, Stock and coworkers [8,9,15] bases its dosimetry calculations in the operating room after the patient has been positioned and needles inserted. It relies heavily on rapid software calculations, and optimal dosimetry is decided only then and executed in real-time. Identical B&K ultrasound unit (Bruel & Kjaer 3553, Gentofte, Denmark) was used for all patients as well as dedicated software (Varian Medical Systems, Inc., Palo Alto, CA). Both patient groups received the same postoperative care (1 month α-blockers as default) and follow-up.

### Dose equations

BED calculations were performed using exactly the same equations described by Stock et al. [16]. Prostate contours on the 1-month postimplant CT drawn by our team physicists were used to calculate prostate dose–volume histograms (DVH). Clinical target volume (CTV) was equal to the planning target volume (PTV) [17]. To compare doses between implant alone and combined implant with EBRT, BED equations were used. The linear-quadratic model was used to determine the BED for EBRT treatments using the equation:

(1) $$\mathrm{BED}=\mathrm{nd}\left[1+\left(d/\alpha /\beta \right)\right]$$

where n = number of fractions = 25; d = dose per fraction = 1.8 Gy; and α/β = a tissue and effect specific parameter associated with the linear-quadratic model = 2. The equation used to calculate the BEDs for the low-dose-rate permanent decaying implants with ^125^I was:

(2) $$\mathrm{BED}=\left(R_{0}/\lambda \right)\left\{1+\left[R_{0}/\left(\mu +\lambda \right)\left(\alpha /\beta \right)\right]\right\}$$

where R_0_ = initial dose rate of implant = (D90)(λ); λ = radioactive decay constant = 0.693/T_1/2_; T_1/2_ = radioactive half-life of isotope ^125^I = 59.8 days; μ = repair rate constant = 0.693/t_1/2_; and t_1/2_ = tissue repair half-time = 1 h.

### Statistical analyses

Baseline characteristics are reported as percentage for dichotomous variables, and as mean, standard deviation (SD), median, range, and estimated 95% confidence interval (CI) for continuous variables. Mann–Whitney U test was used for comparison of two non-parametric means; χ^*2*^ test was used for two proportions. Bivariate Spearman’s correlations were obtained for variables potentially appropriate for multivariate analysis. We used survival- analysis techniques for comparison between PP and IoP study groups. Estimated likelihood of biochemical failure was calculated for each group by the Kaplan-Meier product limit estimation. The log-rank test was used to compare differences between curves. Multivariate Cox proportional hazards model was used to evaluate the relationship between clinical and pathological variables and biochemical failure. The independent variables were treatment technique, age, PSA levels, Gleason score, T clinical stage, ADT, and dosimetry measures. Adjusted hazard ratios (HR) and their 95% CI were computed. All tests were two-tailed, and statistical significance was defined as *P* value < 0.05. The analyses were performed using the PASW Statistics 19.0 for Windows (SPSS Inc., Chicago, IL).

## Results

### Patients’ characteristics

Characteristics of the 1176 patients comprising the cohort are listed in Table 1. The median follow-up for the entire cohort was 47 months (range, 1–155); the median follow-up was 83 months (range, 3–155) for the PP group and 47 months (range, 1–132) for the IoP group. Patients who received brachytherapy using the PP technique were slightly older (1 year in median) with higher pretreatment PSA levels (1 ng/ml in median) and disease stage; higher percentage of intermediate risk group, but with lower Gleason sum scores and with smaller prostate volume (*P* < 0.01).

**Table 1:** Baseline clinical characteristics of study patients according to implant technique

**Characteristics**	**Total sample**	**Implant technique**		***P *** **value**
		**PP**	**IoP**	
	**(n =1176)**	**(n =132)**	**(n = 1044)**	
Age, years				
Mean (SD)	66.2 (6.5)	67.8 (5.9)	66.0 (6.5)	
Median	67.0	68.0	67.0	
Range	45.0–80.0	50.0–79.0	45.0–80.0	
95% CI	65.9–66.6	66.8–68.8	65.6–66.4	0.002^*^
PSA, ng/ml				
Mean (SD)	7.4 (3.1)	8.6 (3.8)	7.2 (3.0)	
Median	6.7	7.6	6.6	
Range	0.6–22.0	0.7–20.0	0.6–22.0	
95% CI	7.2–7.6	7.9–9.2	7.1–7.4	<0.001^*^
PSA, n (%)				
≤ 10	980 (83.3)	94 (71.2)	886 (84.9)	
> 10	196 (16.7)	38 (28.8)	158 (15.1)	<0.001^**^
Gleason score,				
Mean (SD)	5.9 (0.6)	5.3 (0.8)	6.0 (0.5)	
Median	6.0	5.0	6.0	
Range	3.0–7.0	3.0–7.0	3.0–7.0	
95% CI	5.91–5.98	5.2–5.5	5.9–6.1	<0.001^*^
Gleason score < 7, n (%)	1044 (88.8)	128 (97.0)	916 (87.7)	0.002^**^
Prostate volume, ml				
Mean (SD)	38.4 (9.6)	35.0 (9.1)	38.8 (9.6)	
Median	38.0	35.0	39.0	
Range	10.0–74.0	13.0–57.0	10.0–74.0	
95% CI	37.8–68.9	33.4–36.6	38.2–39.4	<0.001^*^
Clinical stage, n (%)				
T_1_-T_2_a	1086 (92.3)	113 (85.6)	973 (93.2)	
> T_2_a	56 (4.8)	14 (10.6)	42 (4.0)	0.001^**^
ADT, n (%)	442 (37.6)	54 (40.9)	388 (37.2)	0.4^**^
EBRT, n (%)	132 (11.2)	4 (3)	128 (12.3)	0.002^**^
Follow-up, months				
Mean (SD)	51.8 (35.1)	80.5 (43.5)	48.1 (32.1)	
Median	47.0	83.0	47.0	
Range	1.0–155.0	3.0–155.0	1.0–132.0	
95% CI	49.7–53.8	72.9–87.9	46.2–50.1	<0.001^*^

*Mann–Whitney test, α < 0.05; **Chi-square test, α < 0.05.

PP, Preplanning; IoP, Intraoperative planning; PSA, Prostate-specific antigen; ADT, Androgen deprivation therapy; EBRT, External beam radiation.

### Oncological endpoints in the entire cohort

Of all 1176 patients, 66 had evidence of biochemical relapse, 38 were lost to follow-up, and 83 died of non-prostate cancer causes. During the follow-up only one patient died from prostate cancer. The 5- and 7-year biochemical control rates were 95% and 90%, respectively, for the entire cohort.

Characteristics used for multiple regression analyses to correlate with biochemical failure were: age, pretreatment PSA level, Gleason score, clinical T stage, administration of neoadjuvant ADT and implant technique IoP vs. PP. EBRT was not included since it was given only to patients with Gleason 7.

The multivariate Cox regression analyses identified PSA, ADT and implant technique as independent prognostic factors for biochemical failure (Table 2, Model 1). Implant technique was the strongest independent prognostic factor (HR = 0.13, IoP vs. PP).

**Table 2:** Multivariate Cox regression analyses for biochemical failure in the entire cohort (n = 1176)

**Factor**	**Model 1**		**Model 2**	
	**Hazard ratio (95% CI)**	**p-value**	**Hazard ratio (95% CI)**	**p-value**
Age	0.99 (0.94–1.03)	0.49	1.01 (0.95–1.07)	0.78
PSA	1.09 (1.02–1.17)	0.011	1.08 (0.96–1.22)	0.22
Gleason	1.02 (0.72–1.46)	0.90	1.55 (0.73–3.27)	0.25
Clinical T stage	1.32 (0.59–2.94)	0.49	1.64 (0.48–5.55)	0.43
ADT	0.55 (0.31–0.99)	0.045	0.20 (0.07–.057)	0.002
Technique	0.13 (0.07–0.23)	<0.001	-----------	
BED	-----------		0.987 (0.981–0.993)	<0.001

PSA, Prostate-specific antigen; ADT, Androgen deprivation therapy; BED, Biological effective dose.

**Figure 1:**
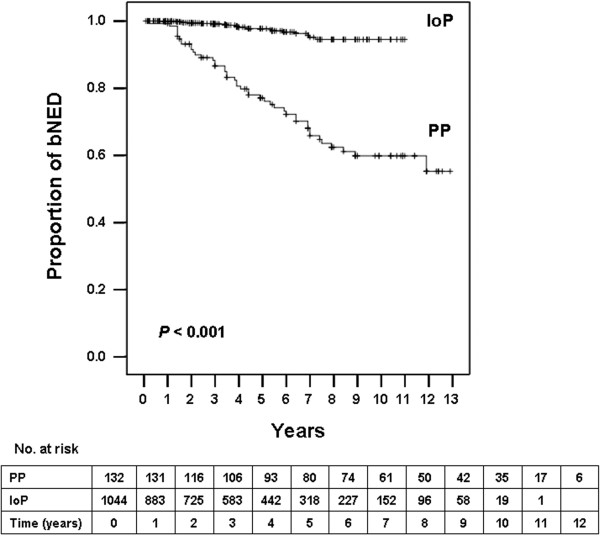
**Kaplan-Meier survival curves for patients with biochemical no evidence of disease (bNED) of the whole cohort.** Number of patients at risk is shown against each time interval. PP, preplanning; IoP, intraoperative planning.

The 5- and 7-year actuarial biochemical control stratified by implant technique was 78% and 67% for the PP group and 99% and 95% for the IoP group, respectively (Figure 1).

The 5- and 7-year actuarial biochemical control stratified by baseline PSA ≤ 10 or > 10 ng/ml was 82% and 74% or 68% and 50% (*P* = 0.97) for the PP group, and, 98% and 95% or 97% and 93% (*P* = 0.97) for the IoP group, respectively (*P* < 0.001, between the groups).

**Figure 2:**
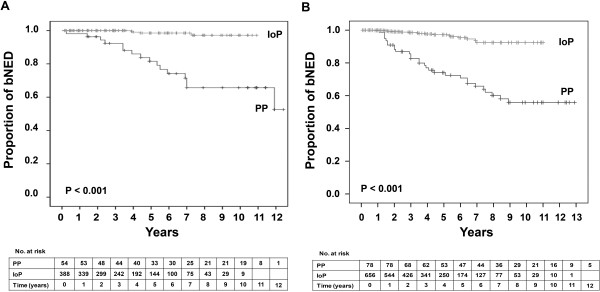
**Kaplan-Meier survival curves for patients with biochemical no evidence of disease (bNED) of the whole cohort who did (A) or did not (B) receive androgen deprivation therapy.** Number of patients at risk is shown against each time interval. PP, preplanning; IoP, intraoperative planning.

The 5- and 7-year actuarial biochemical control stratified by patients who received or did not receive ADT was 82% and 66% or 75% and 66% (*P* = 0.41) for the PP group, and, 99% and 98% or 98% and 95% (*P* = 0.63) for the IoP group, respectively, (Figure 2).

The difference in the postimplant dosimetry presented as BED between the two methodologies was strikingly significant (Table 3). Therefore, the implant technique was replaced by BED in the multivariate Cox regression analysis, which identified ADT and BED as independent prognostic factors for biochemical failure (Table 2, Model 2).

**Table 3:** BED according to implant technique

			**Implant technique**		***P *** value^*^
			**PP**	**IoP**	
**Total sample:** Brachytherapy & brachytherapy combined with EBRT	**n**	**1176**	**1 32**	**1044**	
	**BED, Gy**				
	Mean (SD)	193 (28)	70 (20)	197 (16)	
	Median	195	82	196	
	Range	35–267	35–123	122–267	
	95% CI	191–195	74–86	196–198	<0.001
**Brachytherapy only**	**n**	**1033**	**128**	**905**	
	**BED, Gy**				
	Mean (SD)	189 (25)	80 (19)	194 (13)	
	Median	193	82	194	
	Range	35–250	35–123	122–250	
	95% CI	187–191	73–86	193–195	<0.001

*Mann–Whitney test.

PP, Preplanning; IoP, Intraoperative planning; BED, Biological effective dose; EBRT, External beam radiation.

### Oncological endpoints in the brachytherapy monotherapy group

We further analyzed the cohort of patients by excluding those who received brachytherapy combined with EBRT leaving 1033 patients as monotherpay group (PP, 128 and IoP, 905). The difference in the postimplant dosimetric measures between the two methodologies was also strikingly significant (Table 3). To exclude the possibility that higher BED values in the IoP monotherapy group were a result of increased amounts of implanted ^125^I, we calculated the activity of ^125^I implanted per 1 cc of prostate. The mean amount of ^125^I activity was 0.97 ± 0.15 mCi/cc in the PP group compared to 0.87 ± 0.13 mCi/cc in the IoP group (*P* < 0.001) indicating that the PP group received clinically similar mCi amounts as the IoP group. The 5- and 7-year actuarial biochemical control rate in the monotherapy group was 77% and 70% for the PP group compared to 97% and 95% for the IoP group, respectively.

### Oncological endpoints in the first 132 consecutive patients of each group

To reduce potential bias because of differences in the median follow-up period and the number of patients between the groups we created a subsample composed from all patients (132) receiving PP and first 132 consecutive patients who received IoP. This subsample analysis is not restricted for healthier participants and minimize systematic (time- dependent confounding) bias. As shown in Table 4, the characteristics of the groups were similar. The multivariate Cox regression analyses in this sub-group identified again implant technique as a strong independent prognostic factor for biochemical failure (HR = 0.19, 95% CI 0.09-0.42).

**Table 4:** Baseline clinical characteristics of first 132consecutive study patients from each technique group

**Characteristics**	**Total sample**	**Implant technique**		***P *** **value**
		**PP**	**IoP**	
	**(n =264)**	**(n =132)**	**(n = 132)**	
Age, years				
Mean (SD)	67.7 (5.8)	67.9 (6.0)	67.4 (5.7)	
Median	68.0	68.0	68.0	
Range	50.0–83.0	50.0–83.0	52.0–78.0	
95% CI	66.9–68.4	66.9–68.9	66.4–68.4	0.6^*^
PSA, ng/ml				
Mean (SD)	8.2 (3.4)	8.6 (3.7)	7.8 (3.1)	
Median	7.6	7.6	7.5	
Range	0.7–20.0	0.7–20.0	0.8–17.0	
95% CI	7.8–8.6	7.9–9.2	7.3–8.3	0.1^*^
PSA, n (%)				
≤ 10	201 (76.1)	94 (71.2)	107 (81.1)	
> 10	63 (23.9)	38 (28.8)	25 (18.9)	0.06^**^
Gleason score,				
Mean (SD)	5.5 (0.9)	5.3 (0.9)	5.7 (0.8)	
Median	6.0	5.0	6.0	
Range	1.0–7.0	2.0–7.0	1.0–7.0	
95% CI	5.4–5.6	5.1–5.4	5.6–5.8	<0.001^*^
Gleason score < 7, n (%)	248 (93.9)	128 (97)	120 (90.9)	0.07^**^
Clinical stage, n (%)				
T_1_-T_2_a	225 (90.7)	113 (89.0)	112 (92.6)	
> T_2_a	23 (9.3)	14 (11.0)	9 (7.4)	0.3^**^
ADT, n (%)	112 (42.4)	54 (40.9)	58 (43.9)	0.6^**^
EBRT, n (%)	16 (6.1)	4 (3)	12 (9.1)	0.07**
Follow-up, months				
Mean (SD)	83.3(39.6)	80.5 (43.5)	86.2 (35.2)	
Median	84.0	83.0	95.5	
Range	3.0–155.0	3.0–155.0	7.0–132.0	
95% CI	78.5–88.1	72.9–87.9	80.2–92.3	0.4^*^

* Mann–Whitney test; ** Chi-square test.

PP, Preplanning; IoP, Intraoperative planning; PSA, Prostate-specific antigen ADT, Androgen deprivation therapy; EBRT, External beam radiation.

**Figure 3:**
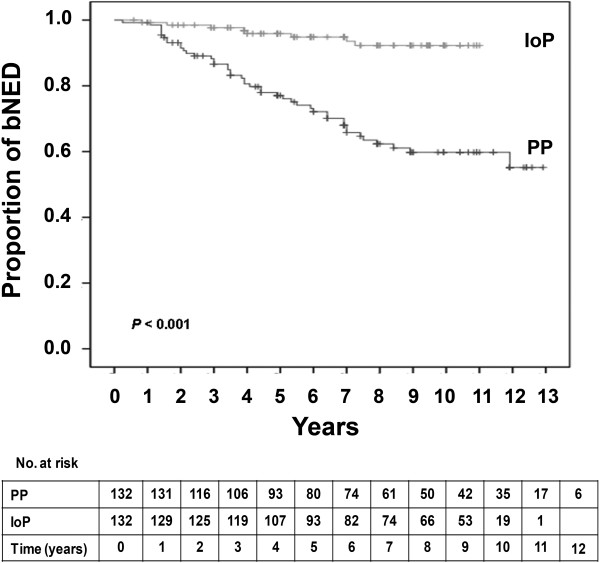
**Kaplan-Meier survival curves for patients with biochemical no evidence of disease (bNED) of the first 132 consecutive patients from each group.** Number of patients at risk is shown against each time interval. PP, preplanning; IoP, intraoperative planning.

The 5- and 7-year actuarial biochemical control rate in this IoP sub-group was 96% and 94%, respectively, and still was significantly higher than in the PP group (Figure 3). When this sub-group was stratified by PSA or ADT, the 5- and 7-year actuarial biochemical control rates were similar to those obtained by the entire IoP group.

## Discussion

In the last three decades brachytherapy was shown to be an equivalent treatment option to other well accepted modalities [18,19]. This development was in part a result of better ultrasonography, better seed placement including coverage of periprostatic disease and certainly better and faster computation capabilities permitting real-time IoP implantation and dosimetry techniques [6]. These improvements were stepwise achieved and the transition from preoperative to intraoperative computation and seed placement dosimetry calculation and on-site rectification if necessary, were only slowly implemented [3,7].

It was less than a decade ago that Woolsey et al. wrote that there were no published results backing up one implant technique over the other [7]. Actually, most brachytherapists switched to various IoP methods based on intuition and not on evidence-produced by prospective examination of the two. Our unique situation where the same brachytherapy team switched from PP to IoP methodology without changing any selection parameters (e.g. age, prostate size, PSA values, clinical stage and Gleason scores) or treatment boundaries enabled such a prospective comparison. In previous publications we have shown that any improvement in results could not be attributed to the learning curve [10,11], and thus our observations are valid ones.

The use of the additional 45 Gy external radiation among the Gleason 7 cohort of patients did not rectify the deeds in the operating room. The comparison between the combination treatments among those treated with PP vs. the IoP, while all received the same 45 external boost and the short term ADT demonstrated again the superiority of the latter. No statistical calculations could be made due to the small number of cases in the PP group. However, one of 4 men in the PP group receiving combination therapy did show biochemical recurrent disease, while none of the 128 men in the IoP group did recur.

In multivariate analyses, seed placement methodology as evidenced by prostate dosimetry parameters such as BED was the strongest one to predict clinical PSA based outcome.

While D90 was shown by many to be of importance [20] and that there is a dose–response for biochemical failure based on day 30 D90 dosimetry [21], it was never demonstrated in a prospective comparison that the two techniques vary so much in their clinical outcome and that dosimetry based differences could predict outcome results pointing to the clear superiority of IoP technique. Potential explanations for this discrepancy in results is the difficult task to reconstitute the exact patient and prostate spatial position in the PP technique, as opposed to real-time dosimetry calculation and implant in the IoP technique. The fact that we limited ourselves in dose calculations to CTV = PTV may in part explain results of this comparative study. Difficulties in reconstituting exact prostate position and shape may affect more the dosimetric outcome in the PP since we did not allow additional margins to prostate boundaries [17].

Evidence-based treatment algorithms regarding the inclusion of short term androgen deprivation in prostate brachytherapy have not been adequately formulated and have been basically extrapolated from our knowledge of their place in external beam regimens. The current appreciation that ADT, be it even for several months duration bears along short and long-term side effects, puts the role of ADT under scrutiny even more [22]. Lee’s et al. [23] observation that implant quality is of greater importance than ADT regarding input of biochemical PSA based control, were seen by us even in the low to intermediate risk patients, as the significance values of the multivariate analysis among both the entire cohort of men are more pronounced for the technique than for ADT.

Our study limitation is firstly, the lack of randomization between the two techniques. Given the fact that most brachytherapists are currently using IoP modalities and will not agree to participate in such a randomized study we believe our results are the best one could get before completely abandoning the PP practices. It should be taken into account that the PP technique was used on 132 patients before we switched to the IoP, which may have had influenced outcome. Although our previous reports as well as the present analysis of the 132–132 patients (Table 4) have not supported this notion [10,11]. Secondly, our study lacks calculations which include an additional margin to prostate gland, the lack of which may explain some of the profound dosimetric differences observed between the two methodologies. Our results should be looked at as a single center experience with perhaps some limits in projecting results on other centers. However, the fact that the current trend of other centers was to switch to the IoP methodology attests to the fact that their results were similar.

We have produced and communicated the entire gamut of data we believe is necessary to build the case in favor of the IoP methodology going from 30 days dosimetry significantly better results [10], to 24 months urinary morbidity similar outcomes [5], to mid-term PSA kinetics showing quicker and more profound PSA declines [11], to the current PSA-based clinical outcomes and far better bNED rates.

## Conclusions

Our long-term actuarial biochemical results add the necessary support to the current literature: modern intraoperative calculation based brachytherapy whether given as monotherapy for low risk disease or in combination with EBRT among Gleason 7 intermediate risk disease is an excellent treatment modality for localized low to intermediate risk prostate cancer patients.

## Abbreviations

bNED: biochemical no evidence of disease; PP: Preoperative planning; IoP: Real-time intraoperative planning; ADT: Androgen deprivation therapy; BED: Biological effective dose; CT: Computerized tomography; EBRT: External beam radiotherapy; PSA: Prostate-specific antigen; D90: Values of the minimal dose received by 90% of the prostate volume; TRUS: Transrectal ultrasound; ABS: American Brachytherapy Society; DVH: Dose-volume histogram; CTV: Clinical target volume; PTV: Planning target volume; SD: Standard deviation; CI: Confidence interval; HR: Hazard ratio; Gy: Gray.

## Competing interest

The authors declare that they have no competing interests.

## Authors’ contributions

HM and NJM conceived of the study, and participated in its design and coordination and wrote the manuscript. JC has made substantial contributions to analysis and interpretation of data. LG performed the statistical analysis. All authors read and approved the final manuscript.
